# Influence of c-Src on hypoxic resistance to paclitaxel in human ovarian cancer cells and reversal of FV-429

**DOI:** 10.1038/cddis.2017.367

**Published:** 2018-01-11

**Authors:** Qinglong Guo, Lu Lu, Yan Liao, Xiaoping Wang, Yi Zhang, Yicheng Liu, Shaoliang Huang, Haopeng Sun, Zhiyu Li, Li Zhao

**Affiliations:** 1 State Key Laboratory of Natural Medicines, Jiangsu Key Laboratory of Carcinogenesis and Intervention, China Pharmaceutical University, 24 Tongjiaxiang, Nanjing 210009, People’s Republic of China; 2 Jiangsu Key Laboratory of Drug Design and Optimization, Department of Medicinal Chemistry, China Pharmaceutical University, 24 Tongjiaxiang, Nanjing 210009, People’s Republic of China; 3 State Key Laboratory of Natural Medicines, Department of Natural Medicinal Chemistry, China Pharmaceutical University, 24 Tongjiaxiang, Nanjing 210009, People’s Republic of China

## Abstract

SRC family kinase was documented to have vital roles in adjusting cancer cell malignant behaviors. To date, the role of c-Src, a member of SRC family kinase, in resistance to paclitaxel in human ovarian cancer cells under hypoxia has not been investigated. In the present study, we discovered that hypoxic environment suppressed paclitaxel-induced G2/M phase arrest and blockade of c-Src improved ovarian cancer cells’ sensitivity to paclitaxel. FV-429, a derivative of natural flavonoid wogonin, could suppress gene expression and activation of c-Src, followed by deteriorated Stat3 nuclear translocation and its binding to HIF-1*α*, resulting in paclitaxel resistance reversal through G2/M arrest potentiation. Our study demonstrated that c-Src contributed to hypoxic microenvironment-rendered paclitaxel resistance in human epithelial ovarian cancer cells by G2/M phase arrest deterioration, and through c-Src suppression, FV-429 was capable of reversing the resistance by blocking c-Src/Stat3/HIF-1*α* pathway.

In solid tumor, cells in hypoxic region becomes resistant to chemotherapy and radiotherapy, the reason for which is that hypoxia helps cancer cells survive by inducing several genes involved that accelerate the progression of malignancy.^1^ Physiological hypoxia occurs when the oxygen tension falls below 2%, thus a cultivating environment containing 1% oxygen is commonly used to mimic hypoxic environment *in vitro*.^2, 3, 4, 5^ Ovarian cancer is a common gynecological cancer and one of the lethal cause of death from gynecological malignancy;^6^ however, failure or relapse always exists due to the emergence of constant resistance. It has been reported that around 69% ovarian tumors from patients overexpress hypoxia inducible factor-1*α* (HIF-1*α*),^7, 8^ indicating that hypoxia may be closely related to ovarian tumors. Study showed that hypoxia increased G0/G1 phase percentage in ovarian cancer cells.^9^ However, the effect of paclitaxel is based on the inhibition of microtubule depolymerization, leading to G2/M phase arrest in cancer cells, which can be weakened by the effect induced by hypoxia. Considering on the uncertainty, the resistance to paclitaxel is probably due to G1 phase promotion rendered by hypoxic environment. However, the inner mechanism of the regulation on this cell cycle arrest still remains unclear.

Non-receptor tyrosine kinase c-Src seems to be associated with hypoxia.^1, 10, 11, 12^ Signal transducer and activator of transcription 3 (Stat3), a transcription factor having vital roles in malignancy, can be activated by c-Src.^13^ Mccann *et al.*^14^ found that Stat3 activation contributed to hypoxia-induced resistance. Moreover, Huang *et al.*^9^ found that blocking HIF-1*α* can improve G2/M arrest induced by paclitaxel under hypoxia. Thus we supposed that c-Src/Stat3/HIF-1*α* axis may regulate hypoxic resistance to paclitaxel under hypoxia.

FV-429, a derivative of wogonin, which is one of the main components extracted from *Scutellaria baicalensis Georgi*, exerted various anticancer activities.^15^ It was optimized with bis (2-hydrocyethyl) amino propoxy substitution at C_7_ position based on wogonin.^16^ FV-429 has been reported to induce apoptosis of HepG2, MDA-MB-231, BGC-823 and MGC-803 cells.^16, 17, 18^ However, its ability to reverse drug resistance has not been investigated.

In our present study, we investigated the reversal of FV-429 on hypoxic resistance to paclitaxel on human epithelial ovarian cancer cells and the mechanism of this resistance associated with c-Src axis regulation.

## Results

### Hypoxia deteriorated G2/M arrest induced by paclitaxel

**Figure 1:**
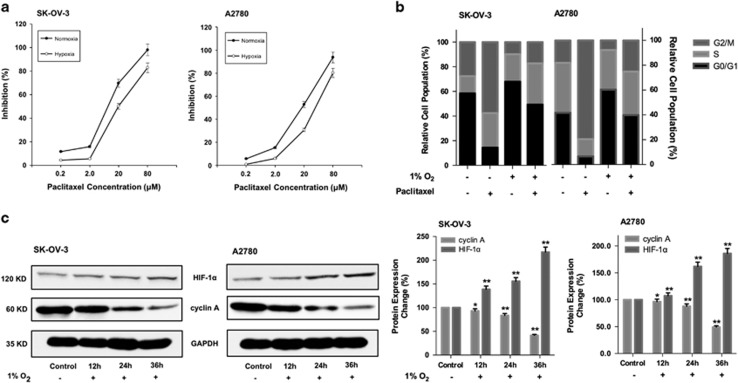
Hypoxia deteriorated G2/M arrest induced by paclitaxel. (**a**) Cell growth inhibition of paclitaxel treatment under normoxia and hypoxia assessed by MTT assay. Data had been statistically analyzed by Microsoft Excel 2013 and expressed as means±S.D. for three independent experiments. (**b**) The influence of paclitaxel treatment on cell cycle under normoxia and hypoxia. Summary of the percentage of cells at G0/G1, S and G2/M phase was performed. (**c**) After 12, 24 and 36 h hypoxic incubation, cyclin A and HIF-1*α* expression detected by western blottings. Protein expression change was represented by densitometric analysis. The results are representative of three independent experiments and expressed as means±S.D., *P<0.05 and **P<0.01, compared with the control groups

Hypoxic culture condition was used to mimic the hypoxic environment in solid tumor. The resistance index (RI) was utilized as an item to access the resistance of cancer cells to drugs. It was considered resistant when RI was >2.0.^19^ According to Figure 1a, IC_50_ was calculated according to cell growth inhibition. The IC_50_ of paclitaxel in hypoxic condition (18.0±0.9 *μ*M) was significantly higher than that in normoxic condition (5.1±0.9 *μ*M) on SK-OV-3 cells, and the same phenomenon was exerted on A2780 cells (28.5±0.8 *μ*M *versus* 8.3±0.9 *μ*M). The RI was 3.5 on SK-OV-3 cell line and 3.4 on A2780 cell line. Cell cycle analysis was conducted to investigate if the resistance was related to cell cycle. As shown in Figure 1b, cell population at G2/M phase induced by paclitaxel of hypoxia groups was much smaller than that of the normoxia groups. Moreover in Figure 1c, during longer periods under hypoxia (12, 24, 36 h), cylin A, which was expressed at the end of G1 phase till the end of G2 phase and reached the highest during G2 phase,^20^ was downregulated.

### c-Src contributed to the resistance to paclitaxel under hypoxia

**Figure 2:**
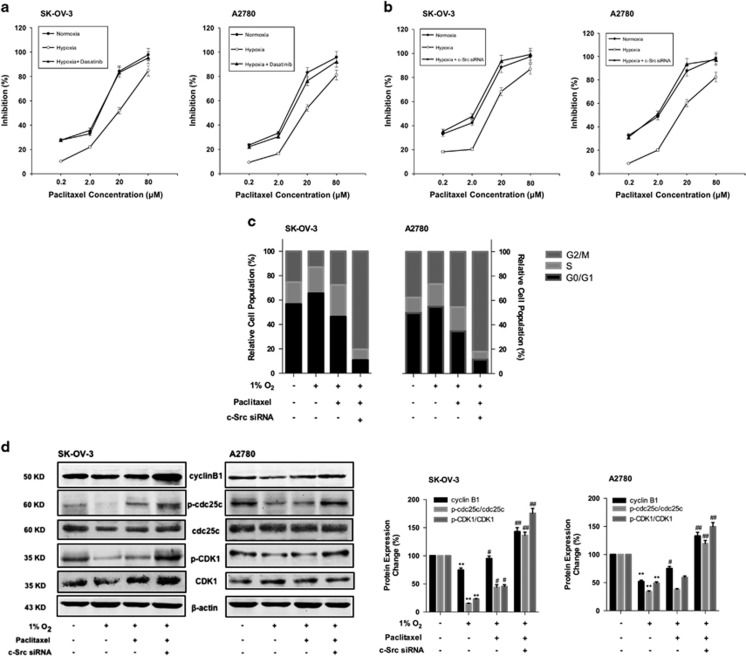
c-Src contributed to the resistance to paclitaxel under hypoxia. (**a**) The cell growth inhibition of paclitaxel combined dasatinib assessed by MTT assay. Data had been statistically analyzed by Microsoft Excel 2013 and expressed as means±S.D. for three independent experiments. (**b**) Cell growth inhibition of paclitaxel on c-Src siRNA transfected cells assessed by MTT assay. Data had been statistically analyzed by Microsoft Excel 2013 and expressed as means±S.D. for three independent experiments. (**c**) The influence of c-Src siRNA transfection on cell cycle with paclitaxel treatment under hypoxia. Summary of the percentage of cells at G0/G1, S and G2/M phase was performed. (**d**) The influence of c-Src siRNA transfection on the expression of cylin B1, p-cdc25c, cdc25c, p-CDK1 and CDK1 with paclitaxel treatment under hypoxia. Protein expression change was represented by densitometric analysis. The results are representative of three independent experiments and expressed as means±S.D., **P<0.01, compared with normoxia control groups and ^#^*P*<0.05 and ^##^*P*<0.01, compared with the hypoxia control groups

In order to investigate whether c-Src had an important role as a regulator, we added c-Src inhibitor dasatinib (dose selection indicated in Supplementary Figures S1 And S2) to the system and utilized c-Src small interfering RNA (siRNA) to silence CSK (gene of c-Src), as shown in Figures 2a and b, and the results illustrated that, with c-Src inhibition both at protein and gene levels, the sensitivity of cells to paclitaxel recovered from hypoxia. As hypoxic resistance to paclitaxel was associated with cell cycle, the influence of c-Src on cell cycle was examined. Figure 2c showed that paclitaxel-induced G2/M phase arrest under hypoxia was enhanced when CSK was knocked down. Moreover, in Figure 2d, with blockage of CSK in the paclitaxel-treated groups under hypoxia, the expression of cyclin B1 and the protein expression ratios of p-cdc25c/cdc25c and p-CDK1/CDK1 increased.

### FV-429 enhanced the sensitivity to paclitaxel through G2/M arrest promotion

**Figure 3:**
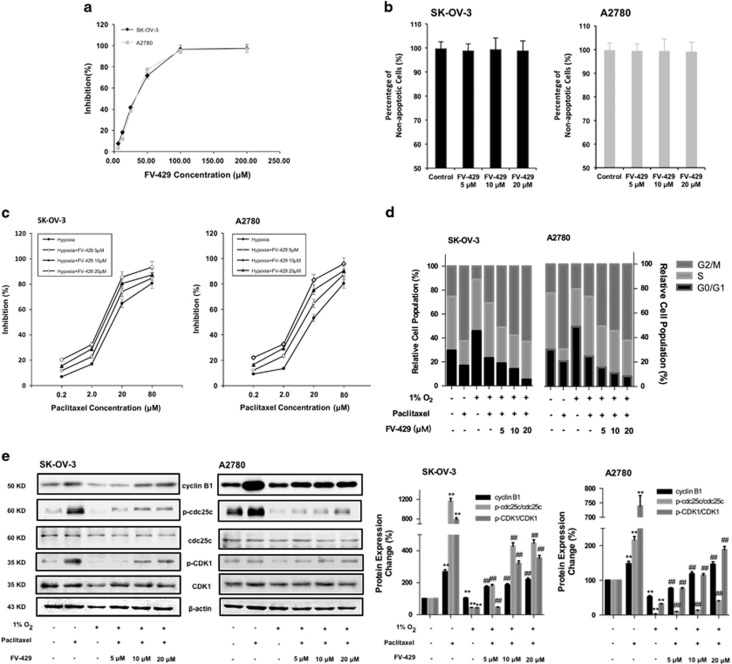
Mild doses of FV-429 enhanced paclitaxel-induced G2/M phase arrest under hypoxia. (**a**) The influence of FV-429 on cell growth inhibition assessed by MTT assay. Data had been statistically analyzed by Microsoft Excel 2013 and expressed as means±S.D. for three independent experiments. (**b**) Percentage of non-apoptotic cells induced by 5, 10 and 20 *μ*M FV-429 detected by Annexin V/PI double staining assay. Data had been statistically analyzed by Microsoft Excel 2013 and expressed as means±S.D. for three independent experiments. (**c**) The influence of FV-429 on cell growth inhibition of paclitaxel under hypoxia assessed by MTT assay. Data had been statistically analyzed by Microsoft Excel 2013 and expressed as means±S.D. for three independent experiments. (**d**) The influence of FV-429 on cell cycle with paclitaxel treatment under hypoxia. Summary of the percentage of cells at G0/G1, S and G2/M phase was performed. (**e**) The influence of FV-429 on the expression of cylin B1, p-cdc25c, cdc25c, p-CDK1 and CDK1 with paclitaxel treatment under hypoxia. Protein expression change was represented by densitometric analysis. The results are representative of three independent experiments and expressed as means±S.D., **P<0.01, compared with normoxia control groups and ^##^*P<*0.01, compared with the hypoxia control groups

In order to investigate the reverse effect of FV-429 on hypoxic resistance to paclitaxel in ovarian cancer cells, proper doses should be chosen primarily. We first assessed the influence of FV-429 on cell viability of SK-OV-3 and A2780. As shown in Figure 3a, doses without severe proliferation inhibition were selected. To confirm that the doses we selected did not induce significant apoptosis, Annexin V/propidium iodide (PI) double staining assay was conducted. In Figure 3b, FV-429 at 5, 10 and 20 *μ*M did not exhibit obvious apoptosis induction after 24 h treatment, with inhibition rates of 0.43, 0.24 and 0.27% on SK-OV-3 and 0.33, 0.14 and 0.35% on A2780. Figure 3c showed that 5, 10 and 20 *μ*M FV-429 enhanced the sensitivity of the cells to paclitaxel under hypoxia in a dose-dependent manner. The data in Figure 3d showed that FV-429, combined with paclitaxel, significantly increased the percentage of the cells at G2/M phase under hypoxia in a dose-dependent manner. Furthermore, the expression of G2/M phase-related protein had been examined, as in Figure 3e, and the expression of cyclin B1 and the ratios of p-cdc25c/cdc25c and p-CDK1/CDK1 were upregulated.

### FV-429 deteriorated c-Src/Stat3/HIF-1*α* pathway under hypoxia

**Figure 4:**
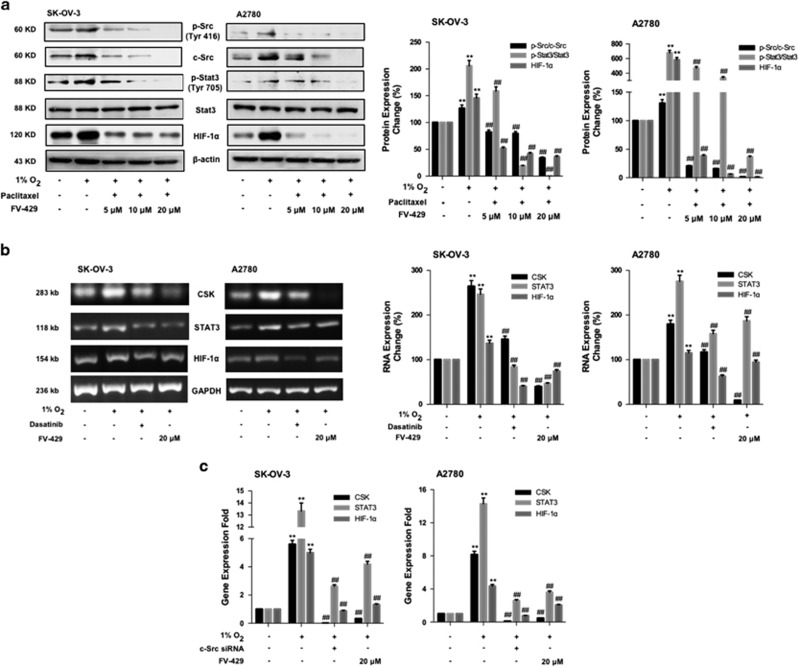
FV-429 suppressed c-Src/Stat3/HIF-1*α* pathway and c-Src mRNA expression. (**a**) The influence of FV-429 on p-Src (Tyr 416), c-Src, p-Stat3 (Tyr 705), Stat3 and HIF-1*α* expression with paclitaxel treatment under hypoxia. Protein expression change was represented by densitometric analysis. The results are representative of three independent experiments and expressed as means±S.D., **P<0.01, compared with normoxia control groups and ^##^*P<*0.01, compared with the hypoxia control groups. (**b**) The influence of FV-429 on mRNA expression of CSK, STAT3 and HIF-1*α* detected by electrophoresis on 3% agarose gel. RNA expression change was represented by densitometric analysis. The results are representative of three independent experiments and expressed as means±S.D., **P<0.01, compared with normoxia control groups and ^##^*P<*0.01, compared with the hypoxia control groups. (**c**) The influence of FV-429 on mRNA expression of CSK, STAT3 and HIF-1*α* detected by reverse transcriptase PCR. Data had been statistically analyzed and displayed in column charts as means±S.D. for three independent experiments by Graphpad Prism 6.0c, **P<0.01, compared with the normoxia control groups and ^##^*P<*0.01, compared with the hypoxia control groups

From the results in Figure 4a, we found that, with higher doses of FV-429, the ratios of p-Src/c-Src and p-Stat3/Stat3 and HIF-1*α* expression were significantly downregulated compared with the hypoxic control groups. From the results, we realized that not only p-Src but also total c-Src was downregulated by FV-429. Thus we supposed that FV-429 might be able to suppress CSK. Comparing Figures 4b and c, FV-429 showed strong inhibition on CSK expression, which had significant difference from the dasatinib-treated groups (*P*<0.01), but less difference with c-Src siRNA-transfected groups (*P*=0.046).

**Figure 5:**
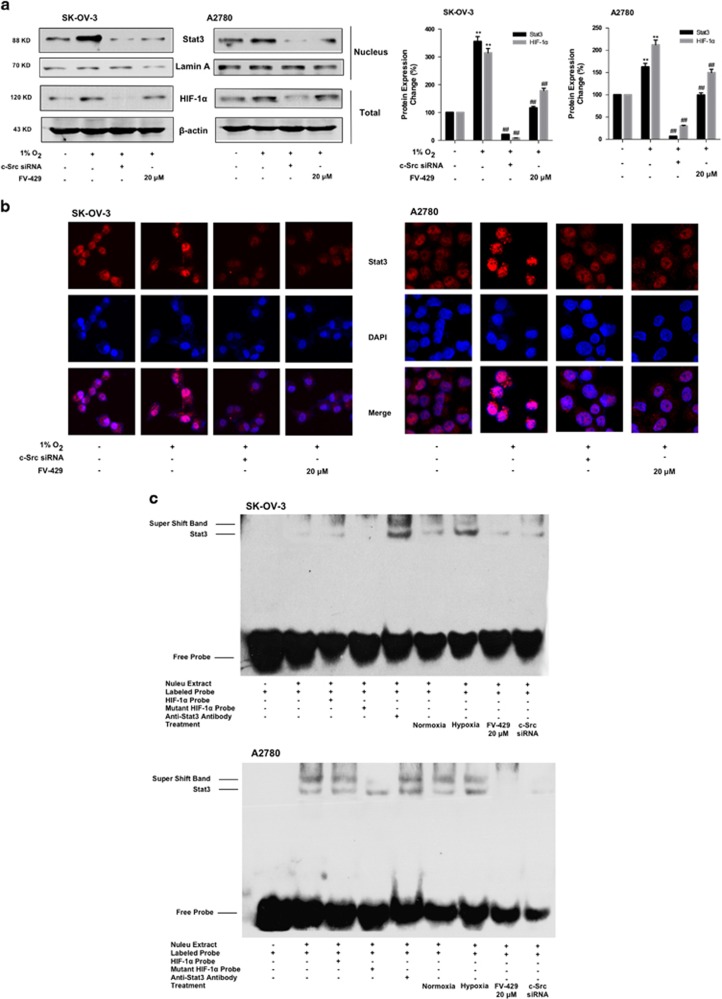
FV-429 inhibited Stat3 nuclear translocation and binding to HIF-1*α*. (**a**) The influence of FV-429 on nuclear expression of Stat3 and total expression of HIF-1*α* under hypoxia. Protein expression change was represented by densitometric analysis. The results are representative of three independent experiments and expressed as means±S.D., **P<0.01, compared with the normoxia control groups and ^##^*P<*0.01, compared with the hypoxia control groups. (**b**) The influence of FV-429 on Stat3 nuclear translocation examined by immunofluorescence (× 600). (**c**) The influence of FV-429 on binding ability of Stat3 to HIF-1*α* under hypoxia assessed by EMSA

Then we further investigated the influence of c-Src on Stat3/HIF-1*α* axis. In Figure 5a, the expression of Stat3 in the nucleus and the total expression of HIF-1*α* were significantly downregulated by FV-429. Furthermore, in Figure 5b, the signal of Stat3 in the nucleus was barely strong in the hypoxia control groups, while the effect was blocked by FV-429. Then we investigated the DNA-binding ability of Stat3. The results of electrophoretic mobility shift assay (EMSA) assay (Figure 5c) showed that FV-429 attenuated the binding ability of Stat3 to HIF-1*α*, which was enhanced in hypoxic condition.

### FV-429 potentiated the effect and G2/M arrest induced by paclitaxel through c-Src/Stat3/HIF-1*α* pathway deterioration *in vivo*

**Figure 6:**
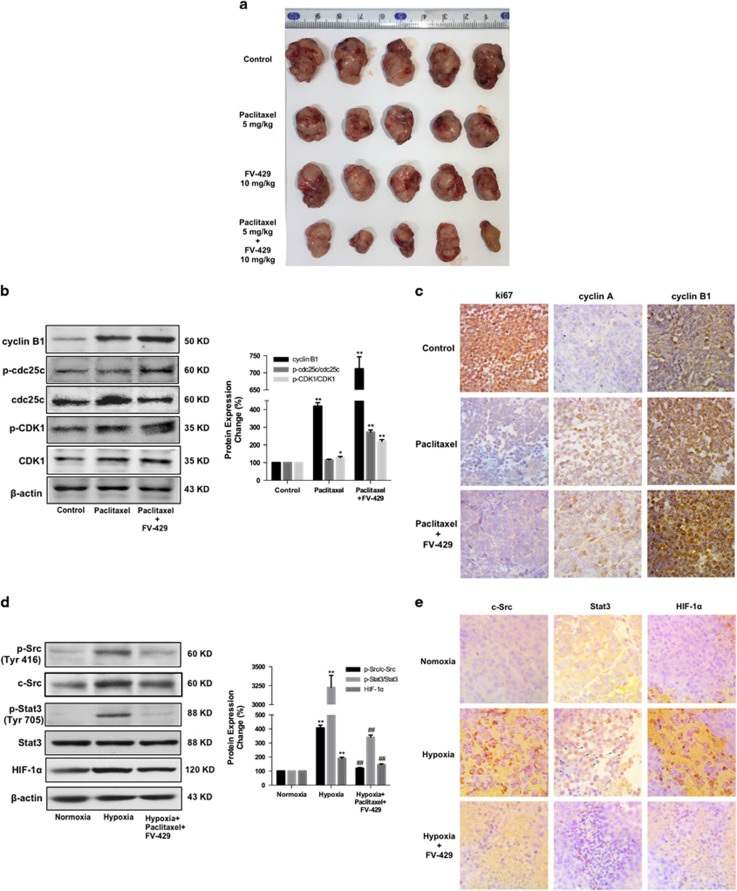
Antixenografted tumor effect and reversal mechanism of FV-429 combined with paclitaxel *in vivo*. (**a**) Image of the resected xenografted tumors. (**b**) Expression of cylin B1, p-cdc25c, cdc25c, p-CDK1 and CDK1 in hypoxic regions of xenografted tumors detected by western blottings. Protein expression change was represented by densitometric analysis. The results are representative of three independent experiments and expressed as means±S.D., *P<0.05 and **P<0.01, compared with the control groups. (**c**) Expression of ki67, cylin A and cylin B1 in hypoxic regions of xenografted tumors detected by immunohistochemistry (× 400). (**d**) Expression of p-Src (Tyr 416), c-Src, p-Stat3 (Tyr 705), Stat3 and HIF-1*α* in normoxic and hypoxic regions of xenografted tumors detected by western blottings. Protein expression change was represented by densitometric analysis. The results are representative of three independent experiments and expressed as means±S.D., **P<0.01, compared with the normoxia control groups and ^##^*P<*0.01, compared with the hypoxia control groups. (**e**) Expression of c-Src, Stat3 and HIF-1*α* in normoxic and hypoxic regions detected by immunohistochemistry (× 400). Arrows referred to Stat3 nuclear translocation

Xenograft mouse model was utilized to assess the effect of combined treatment of mild dose of paclitaxel and FV-429. After treatment with FV-429 (10 mg/kg) and/or paclitaxel (5 mg/kg) for 14 days, the combined group showed stronger effect in tumor growth suppression (Figure 6a). According to the tumor weight analysis, the inhibition rate of 10 mg/kg FV-429 combined with 5 mg/kg paclitaxel group was 57.50%, which was much better than FV-429 single treated group (32.14%) and paclitaxel group (31.06%).

We also confirm the effect of FV-429 on paclitaxel-induced G2/M phase arrest *in vivo*. As shown in Figure 6b, the expression of cylin B1 and the ratios of p-cdc25c/cdc25c and p-CDK1/CDK2 were significantly upregulated in the combined treated group. Moreover, the results of immunohistochemistry (IHC; Figure 6c) suggested that, in the combination group, there were significant increase of cyclin A and cyclin B1 and strong suppression on ki67 nuclear translocation.

Then we confirm the potentiated effect of FV-429 on c-Src/Stat3/HIF-1*α* pathway *in vivo*. As shown in Figure 6d, after FV-429 treatment, the protein expression ratios of p-Src/c-Src and p-Stat3/Stat3 and HIF-1*α* expression decreased in hypoxic tumor tissues (the central part of tumors and normoxic tumor tissues referred to the outer part of tumors^21^), and IHC study (Figure 6e) showed the expression of c-Src and HIF-1*α* and nuclear translocation of Stat3 decreased in the tissues.

### Toxicological assessment

**Figure 7:**
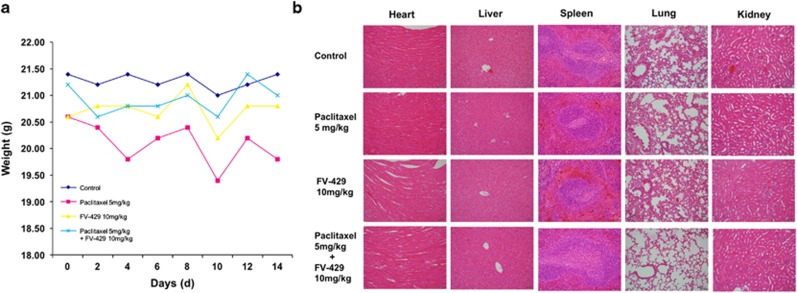
Toxicological assessment. (**a**) Body weight change of xenografted nude mice by days. Data had been statistically analyzed by Microsoft Excel 2013 and expressed as means±S.D. (*n*=5). (**b**) The influence of FV-429 and paclitaxel single or combined treatment on the heart, liver, spleen, lung and kidney was examined by hematoxylin and eosin staining (× 200)

We investigated the influence of FV-429 single or paclitaxel combined treatment on animals’ weight, main organs and peripheral blood. As shown in Figure 7a, there was a significant decrease of animals’ weight of mice in the paclitaxel single group, but in FV-429 single or combined group, the effect was mild. And the results of hematoxylin and eosin staining of the five main organs of an individual showed that both paclitaxel and FV-429 exerted no significant toxicities (Figure 7b). Moreover, paclitaxel treatment resulted in an increase of band neutrophils and decrease of monocytes and total white blood cells, but in FV-429 single or combined groups, the trend was improved (Table 1).

## Discussion

In the present study, we demonstrated that hypoxic condition rendered resistance to paclitaxel in human epithelial ovarian cancer cells through deteriorating paclitaxel-induced G2/M phase arrest. Blockage of c-Src can significantly improve the sensitivity of the cells to paclitaxel under hypoxia and paclitaxel-induced G2/M arrest. Further study revealed that flavonoid compound FV-429 suppressed gene expression and protein activation of c-Src under hypoxic condition. With c-Src suppression by FV-429, the translocation of Stat3 into nucleus and its binding to HIF-1*α* was inhibited, leading to paclitaxel-induced G2/M phase enhancement. Also we demonstrated that FV-429, combined with paclitaxel, exerted better antixenograft tumor effect than both single treated groups *in vivo*, with hematological side effects’ improvement and no toxicity on animals’ main organs.

Among gynecological malignancies, ovarian cancer is the most difficult to cure.^22^ Maximum debulking surgery followed by chemotherapy consisting of paclitaxel and platinum is the standard treatment for advanced ovarian cancer.^23^ Though >70% of the patients responded to chemotherapy initially, the majority relapsed.^22^ Recently, there is growing evidence highlighting the importance of tumor microenvironment-mediated chemoresistance mechanisms in ovarian cancer.^24^ It has long been noted that the rapidly proliferating malignant cells generates greater oxygen consumption, which jointly favor the formation of hypoxic areas within the solid tumors.^25^ Cycling hypoxia is a well-recognized phenomenon within solid tumors that contributes to the resistance to chemotherapy or radiotherapy.^26^ Studies demonstrated that cells in hypoxic regions tend to be arrested at G1 phase.^9, 27, 28^ Paclitaxel was found to stabilize cytoplasmic microtubules, thus the cells would stagnate at the beginning of M phase, followed by induction of apoptosis and/or mitotic catastrophe.^29^ According to our results, the antiproliferative effect of paclitaxel was attenuated under hypoxia (Figure 1a), with IC_50_ increasing around threefolds. And cell cycle analysis showed a decrease of G2/M phase population in the paclitaxel-treated group under hypoxia (Figure 1b). Considering these, we may safely conclude that hypoxic region in ovarian cancer contributed to the resistance by inhibiting paclitaxel-induced G2/M phase arrest.

Abnormal expression or activation of CSK has been documented to have vital roles in tumor malignancy and interacted with mechanisms that regulate paclitaxel sensitivity.^29^ Thus we wondered whether c-Src/Stat3/HIF-1*α* pathway was also aroused in hypoxic resistance to paclitaxel. As we discovered, with both c-Src activation suppression (Figure 2a) and CSK blockage (Figure 2b), the antiproliferative ability of paclitaxel under hypoxia has been greatly improved. Moreover, the cell population at G2/M phase, as well as G2/M phase-specific protein expression, significantly increased (Figure 2c) with CSK silenced (Figure 2d). All these indicated that blockage of c-Src improve ovarian cancer cells’ sensitivity to paclitaxel under hypoxia, thus c-Src may serve as a promising target to overcome hypoxic resistance to paclitaxel in ovarian cancer.

Studies showed that active c-Src promoted HIF-1*α* function by activating Stat3, which bind to and activated HIF-1*α* promoter, resulting in downstream effects.^30, 31^ From the results of Figures 4a and c, Src/Stat3/HIF-1*α* pathway was activated under hypoxia, and the activation was suppressed by FV-429 treatment. From the results, we noticed that not only activated p-Src (phosphorylated at Tyr 416) but also total c-Src decreased. Therefore, we supposed that FV-429 might be able to suppress c-Src gene expression. Comparing the results of Figures 4b and c, the suppression of CSK by FV-429 exhibited less variation with that of c-Src siRNA transfection groups. Besides, with c-Src gene suppression by FV-429, the nuclear translocation of Stat3 has also been blocked (Figures 5a and b), as well as the binding of Stat3 to HIF-1*α* (Figure 5c). And the expression of Hexokinase II and VEGF, both downstream targets of HIF-1*α*, decreased, further indicating the suppression on HIF-1*α* function (Supplementary Figure S3). Moreover, *in vivo* results indicated that FV-429 inactivated c-Src/Stat3/HIF-1*α* pathway in hypoxic tumor tissues (Figures 6d and e) and FV-429, combined with paclitaxel, induced significant increased G2/M-specific protein expression in tumor tissues (Figures 6b and c) with an inhibition rate of 57.5%, confirming the effectiveness of FV-429 in improving paclitaxel treatment by G2/M phase arrest enhancement through c-Src/Stat3/HIF-1*α* pathway *in vivo*.

It has been proposed that chemotherapeutic drugs effectively trigger mitotic catastrophe at low doses, significantly limiting side effects.^32^ The side effect of paclitaxel include allergic reaction, neurotoxicity and so on^33^ but the more common one was hematological toxicity.^34^ As we demonstrated in Table 1, there was a decrease of monocytes and increase of band neutrophils in the paclitaxel single treated group, indicating myelosuppression and immunosuppression existence. However, in groups with FV-429 treatment, the influence was weakened. As it was reported that flavonoids were able to serve as xenobiotics that are metabolized by the cytochrome P-450 enzymes and conjugating protective enzymes,^35^ we speculated that the effect might be attributed to the protective effect of flavonoids. Besides, flavonoids metabolized slowly *in vivo*,^36^ suggesting a longer treating period.

The mechanism we investigated here focused on the influence of c-Src/Stat3/HIF-1*α* pathway regulation on cell cycle. However, whether the mechanism also affects other pharmacological effects induced by paclitaxel, for instance, autophagy, still remained a problem to resolve.

In summary, the present study discovered that c-Src contributed to hypoxic microenvironment-rendered paclitaxel resistance in human epithelial ovarian cancer cells by G2/M phase arrest deterioration, and through c-Src suppression, FV-429 was capable of reversing the resistance by blocking the c-Src/Stat3/HIF-1*α* pathway. Our study provided a new thinking of c-Src suppression in cancer therapy and a promising agent in this field.

## Materials and Methods

### Reagents

FV-429 (MW 429) was prepared by Dr. Zhiyu Li, dissolved in dimethyl sulfoxide (DMSO, Sigma-Aldrich, St. Louis, MO, USA) to 100 mM and stored at −20 °C.

Paclitaxel injection was purchased from Taiji Pharmaceutical Co. Ltd. (Sichuan, China) as stocking solution and diluted with RPMI-1640 (Gibco, Carlsbad, CA, USA) and DMEM (Gibco) medium to a final concentration with organic solute no more than 0.1%. Dasatinib (Sigma-Aldrich) was diluted with DMSO to 100 *μ*M and stored at −20 °C. The drug was freshly diluted with RPMI-1640 and DMEM medium to 0.05 *μ*M before use. MTT (3-(4,5-dimethylthiazol-2-yl)-2,5- diphenytetrazoliumbromide) was obtained from Fluka Chemical Corp. (Ronkonkoma, NY, USA) and was dissolved in 0.01 M phosphate-buffered saline (PBS).

Antibodies against c-Src, HIF-1*α*, Stat3, p-Stat3 (Tyr705), Cyclin B1and GAPDH were products of Bioworld Technology (St. Louis Park, MN, USA). Antibodies against p-Src (Tyr416), p-cdc25c (Ser216) and cdc-25c were products of Cell Signaling Technology (Beverly, MA, USA). Antibodies against Cyclin A, cdc-2 and p-cdc2 (Thr161) were products of Santa Cruz Biotechnology (Santa Cruz, CA, USA). Normal mouse and rabbit IgG-HRB secondary antibody were purchased from Santa Cruz Biotechnology.

### Cell culture

SK-OV-3 (human epithelial ovarian cancer) cells were originally from the Cell Bank of the Shanghai Institute of Biochemistry and Cell Biology, Chinese Academy of Sciences (Shanghai, China). A2780 (human epithelial ovarian cancer) cells were originally from KeyGen Biotechnology (Nanjing, China). SK-OV-3 cells were grown in RPMI-1640, and A2780 were in DMEM; all media contained 10% heat-inactivated fetal bovine serum (Wisent, St. Bruno, Quebec, Canada). All cells were incubated in a humidified atmosphere at 37 °C.

### siRNA transient transfection

C-Src siRNA was purchased from Santa Cruz Biotechnology. SiRNA transfections were performed according to the manufacturer’s instructions using Lipofectamine 2000 reagent (Invitrogen, Carlsbad, CA, USA). After that, the transfected system was removed, and it was not until 24 h cultured in normal media were the cells used for further experiment.

### Normoxic and hypoxic culture conditions

Cells were maintained at 37 °C in humidified incubator containing 20% O_2_, 5% CO_2_ and 75% N_2_ in normoxia. Hypoxic condition were achieved at 37 °C by culturing cells in a modified incubator chamber flushed with a gas mixture containing 1% O_2_, 94% N_2_ and 5% CO_2_ in a humidified atmosphere.

### MTT assay

Cell viability was measured using the MTT assay. Cells (8 × 10^3^) were treated with FV-429 and/or paclitaxel for 24 h at various concentrations. Absorbance (*A*) of the final formazan was measured spectrophotometrically at 570 nm by Universal Microplate Reader EL800 (BioTek Instruments, Winooski, VT, USA). The inhibition ratio (%) was calculated as ((*A*_control_−*A*_treated_)/*A*_control_) × 100%. *A*_treated_ and *A*_control_ are the average absorbance values of three parallel experiments from treated and control groups, respectively. RI was calculated as the IC_50_ of SK-OV-3 and A2780 cells under hypoxia/normoxia.

### Annexin V/PI staining

SK-OV-3 and A2780 cells were harvested and stained with the Annexin V/PI Cell Apoptosis Detection Kit (KeyGen Biotechnology) according to the manufacturer’s instructions. Data were acquired from flow cytometry CellQuest Software (Becton Dickinson (Franklin Lakes, NJ, USA)) and analyzed by FlowJo version 10 (Ashland, OR, USA).

### Cell cycle analysis

SK-OV-3 and A2780 cells were weeded into a six-well plate at 2.5 × 10^5^ cells per well. The cells were incubated for 24 h in serum-free medium and treated with or without FV-429 and/or paclitaxel for another 24 h in normoxia or hypoxia. Then the cells were harvested and fixed in cold 70% ethanol overnight at 4 °C. After washing with PBS, cells were incubated with 100 *μ*l RNaseA (KeyGen Biotechnology) at 37 °C for 30 min. Subsequently, cells were incubated with 400 *μ*l PI (KeyGen Biotechnology) for 30 min at 4 °C in the dark and detected by flow cytometry (Becton Dickinson). Data were analyzed by FlowJo version 10.

### Extraction of cytoplasmic and nuclear fractions

SK-OV-3 and A2780 cells were treated with FV-429 and/or paclitaxel at the indicated concentrations for 24 h. Nuclear and cytosolic protein extract were prepared using a Nuclear/Cytosol Fractionation Kit (BioVision, Mountain View, CA, USA) according to the manufacturer’s protocol. One part of the cytosolic and nuclear extract was used for EMSA. Final detection was performed with western blotting analysis.

### Western blotting analysis

Cells were collected after the operation previously mentioned and then lysed in lysis buffer. Nuclear and cytoplasmic proteins of the cells were extracted as previously mentioned. Protein samples were loaded onto an SDS-PAGE gel and transferred to a nitrocellulose membrane (BioTrace NT; PallCor, Arroyto, Cordoba, Argentina). The membranes were then blocked with 3% fat-free milk in PBST for 1 h and incubated with primary antibodies overnight at 4 °C followed by IgG-HRB secondary antibody for 1 h at room temperature. Detection was performed by X-ray films. All blots were stripped and reprobed with polyclonal anti-GAPDH, anti-*β*-actin or anti-lamin A antibody to ascertain loading of proteins.

### Immunofluorescence confocal microscopy

Treated SK-OV-3 and A2780 cells were harvested and seeded onto glass coverslips processed for immunofluorescence. The glass coverslips were washed twice with cold PBS for 5 min, fixed with 4% paraformaldehyde for 30 min and incubated with 0.3% Triton X-100 for 10 min. After incubation, the cell were blocked with PBS containing with 1% bovine serum albumin for 1 h and incubated with anti-Stat3 antibody at 4 °C overnight. After being washed with PBS for three times, the cells were stained with FITC-conjugated anti-rabbit IgG antibody for 1 h at room temperature. And then the coverslips were stained with diamidinophenylindole for 10 min. The images were captured with an Olympus FV1000 confocal microscope (Tokyo, Japan).

### Electrophoretic mobility shift assay

Nuclear extracts were prepared as described above. According to the manufacturer’s instructions, nonradioactive (biotin label) gel shift assays were performed. In brief, oligonucleotide proves were synthesized, annealed and labeled using the Biotin 3′-END DNA Labeling Kit (Pierce, Waltham, MA, USA). The binding reactions were performed according to the manufacturer’s protocol. Finally, the chemiluminescence of the biotin-labeled DNA was detected using the Chemiluminescent EMSA Kit (Beyotime, Nanjing, Jiangsu, China) and the samples were exposed to X-ray film.

### Analysis of mRNA levels

Cells were harvested after 24 h treatment with dasatinib or FV-429 under hypoxia. Cells that were not treated with the agents were utilized as control group of normoxia or hypoxia. Total RNA was extracted from each sample with Trizol (Takara Biotech, Dalian, China) according to the manufacturer’s instructions. cDNA was synthesized from the isolated RNA by RT and amplified individually by PCR (primer sequences are listed in Table 2).

### Xenografted mouse model

Five-to-6-week-old female BALB/c nude mice were purchased from SLAC laboratory Animals (Shanghai, China) and were raised in air-conditioned rooms with 12 h light per day and fed with standard laboratory food and water. The transplanted tumors were induced by subcutaneous injection into the flanks of the mice with 1.0 × 10^6^ A2780 cells. After 12–14 days, the tumor volume was measured by micrometer calipers, and the mice with similar tumor sizes were randomly divided into four groups with five individuals per group. The mice were treated with 5 mg/kg paclitaxel or/and 10 mg/k paclitaxel every 2 days. After 2 weeks, the mice were killed, and the tumor xenografts were removed and measured. Tumor volume (TV) was calculated using the following formula: TV (mm^3^)=*D*/2 × *d*^2^, where *D* is the longest diameter while *d* is the shortest diameters. This study was approved in SPF Animal Laboratory of China Pharmaceutical University. The animals were weighed every 2 days and monitored for mortality throughout the experimental period to assess toxicity of the treatment.

### Immunohistochemistry

The expression of ki67 (KeyGen Biotechnology), cylin A and cylin B1, as well as c-Src, Stat3 and HIF-1*α*, in tumor tissues of nude mice were assessed as described previously^37^ by goat-anti-mouse antibodies and an Ultra-Sensitive TMSAP Kit (Kit 9710 Maixin, Fuzhou, Fujian, China). All reagents were supplied by Maixin-Bio Co, Fuzhou, China.

### Statistical analysis

Data shown were presented by means±S.D. from triplicate experiments performed in a parallel manner unless otherwise indicated. Statistical analysis was performed using an unpaired two-tailed Student’s *t*-test.

## Figures and Tables

**Table 1:** Hematology profile

**Hematological parameter**	**Control**	**Paclitaxel (5 mg/kg)**	**FV-429 (10 mg/kg)**	**Paclitaxel +FV-429**	**Standard**
White blood cells (× 10^3^ *μ*l)	11.09/12.99	4.06/4.08	6.09/7.52	7.34/8.18	4.5–9.1
Red blood cells (× 10^3^ *μ*l)	10.80/10.55	9.47/9.76	10.15/10.06	11.64/10.17	7.51–16.1
Hemoglobin (g/l)	154/154	145/148	156/139	158/157	126–161
Platelets (× 10^3^ *μ*l)	467/488	472/442	425/502	523/535	115–1037
Band neutrophils (%)	3.54/4.50	18.80/20.40	3.84/5.14	6.50/9.40	0–1
Lymphocytes (%)	91.74/90.60	79.20/78.20	94.94/91.84	87.60/87.40	49–82
Monocytes (%)	4.64/4.74	1.84/1.24	4.34/3.94	5.64/4.04	2–8
Eosinophils (%)	0.14/0.14	0.04/0.24	0.04/0.14	0.14/0.04	0–3
Basophils (%)	0.14/0.14	0.24/0	0.04/0.14	0.14/0.14	0–3
Mean corpuscular volume (fl)	43.3/45.6	46.8/45.7	46.0/44.5	44.8/45.2	41–60
Hematocrit (%)	46.8/48.1	44.3/44.6	46.7/44.8	52.2/46.0	34–50
Mean corpuscular hemoglobin (pg)	14.3/14.6	15.3/15.2	15.4/13.8	15.3/15.4	13–19
Mean corpuscular hemoglobin concentration (%)	329/320	327/332	334/310	341/341	316–354

Two mice of each group were used. Standard ranges were obtained in-house from 100 normal BALB/c nude mice aged 8–12 weeks

**Table 2:** Primer sequences used in the study

**Gene symbol**	**Organism**	**Forward primer (5′→3′)**	**Reverse primer (5′→3′)**
CSK	*Homo sapiens*	TCCGGCCCCGTCTCTCTTGG	ACCCTCACGGGCAGGACAGG
STAT3	*Homo sapiens*	ACCAACAATCCCAAGAATGT	CGATGCTCAGTCCTCGC
HIF-1*α*	*Homo sapiens*	CCTTTGGCTCTTCAGGATGC	GGTCTTTCTGCACATTTGGGTGG
GAPDH	*Homo sapiens*	GGTGTGAACCATGAGTATGACAAC	CCAGTGAGGCAGGGATGATGTTC
