# Supplementary material for: Influence of c-Src on hypoxic resistance to paclitaxel in human ovarian cancer cells and reversal of FV-429
Source: Cell Death Dis. 2018 Jan 11;9(1):e3178–. doi: 10.1038/cddis.2017.367 (PMC5827169; doi:10.1038/cddis.2017.367)
Supplement: Supplementary Material [file cddis2017367x1.pdf]

Influence of c-Src on Hypoxic Resistance to Paclitaxel in Human Ovarian Cancer Cells and Reversal of FV-429

Qinglong Guo[1a], Lu Lu[1a], Yan Liao[a], Xiaoping Wang[a], Yi Zhang[a], Yicheng Liu[a], Shaoliang Huang[a], Haopeng Sun[b], Zhiyu Li[c], Li Zhao[a]*

[1] Contributed equally to this article

[a]State Key Laboratory of Natural Medicines, Jiangsu Key Laboratory of Carcinogenesis and Intervention, China Pharmaceutical University, 24 Tongjiaxiang, Nanjing 210009, People's Republic of China

[b]Jiangsu Key Laboratory of Drug Design and Optimization, Department of Medicinal Chemistry, China Pharmaceutical University, 24 Tongjiaxiang, Nanjing 210009, People's Republic of China.

[c]State Key Laboratory of Natural Medicines, Department of Natural Medicinal Chemistry, China Pharmaceutical University, 24 Tongjiaxiang, Nanjing 210009, People's Republic of China.

*Corresponding author: School of basic medicine and clinical pharmacy, Jiangsu Key Laboratory of Carcinogenesis and Intervention, China Pharmaceutical University, 24 Tongjiaxiang, Nanjing 210009, People's Republic of China

Tel: (+86 025) 83271039

E-mail address: zhaoli@cpu.edu.cn

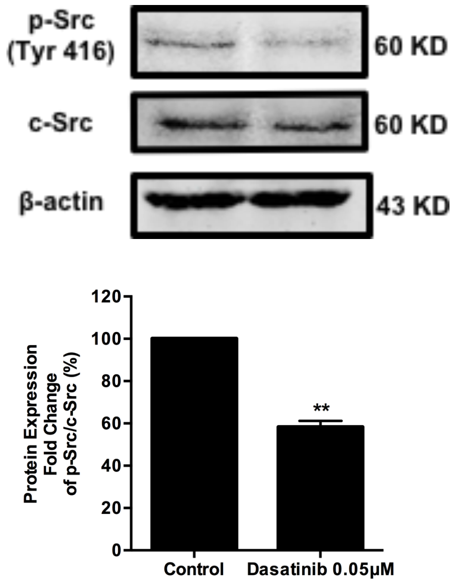

Fig. S1. The influence of dasatinib on c-Src activation of A2780 cells. The expression of c-Src and p-Src (Tyr 416) was detected by western blots after 24 h-treatment. Quantitation had been statistically analyzed by Microsoft Excel 2013 and displayed in column charts as Means ± SD for three independent experiments by Graphpad Prism 6.0c.

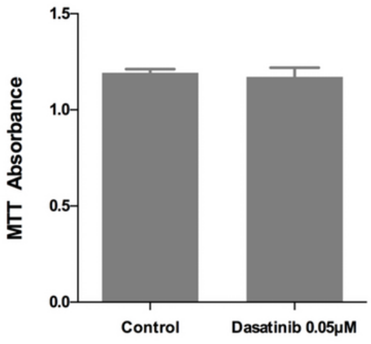

Fig. S2. The influence of dasatinib on MTT absorbance of A2780 cells. MTT absorbance was detected after 24 h-treatment. Data had been statistically analyzed by Microsoft Excel 2013 and expressed as Means ± SD for three independent experiments.

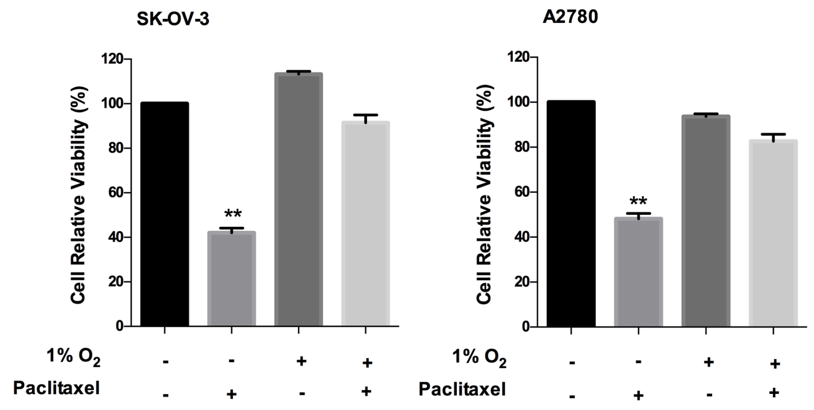

Fig. S3. Influence of paclitaxel treatment on cell viability under normoxia and hypoxia. The effect of 24 h paclitaxel treatment on cell viability in normoxic and hypoxic condition was detected by MTT assay. Data had been statistically analyzed by Microsoft Excel 2013 and displayed in column charts as Means ± SD for three independent experiments by Graphpad Prism 6.0c, ** $p < 0.01$, compared with normoxia control groups.

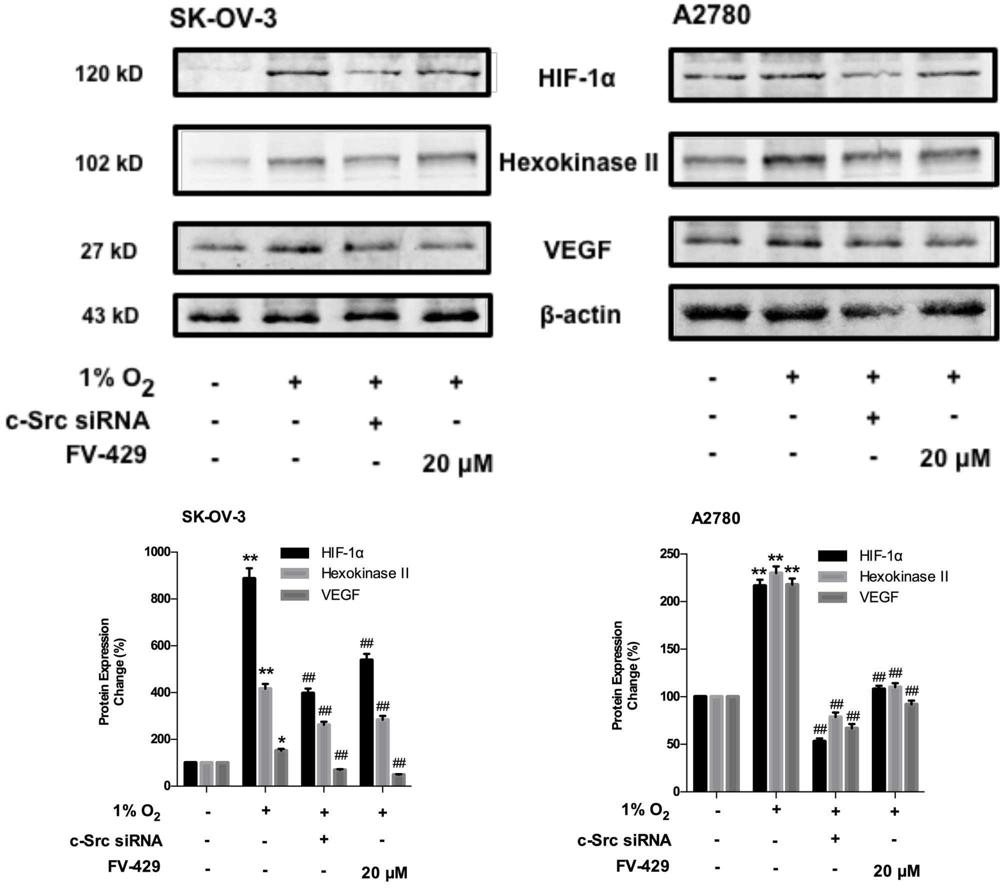

Fig. S4. The influence of FV-429 on HIF-1α, Hexokinase II and VEGF expression under

hypoxia. After 24 h-treatment of FV-429, the expression of HIF-1α, Hexokinase II and VEGF was detected by western blots. Protein expression change was represented by densitometric analysis. The results are representative of three independent experiments and expressed as Means ± SD, ** $p < 0.01$, compared with normoxia control groups, ## $p < 0.01$, compared with hypoxia control groups.

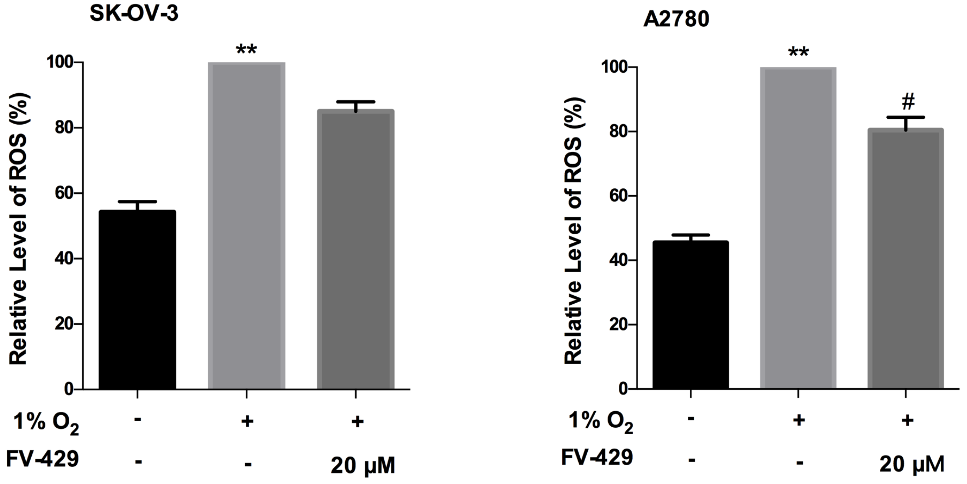

Fig. S5. Influence of FV-429 on intracellular ROS level under hypoxia. After 24 h-treatment with FV-429, the intracellular ROS level was assessed by ROS Detection Kit (KeyGene, Nanjing,China) and detected by flow cytometry. Relative level of ROS was calculated and analyzed by Excel 2013 and expressed as Means±SD for three independent experiments Graphpad Prism 6.0c. ** $p < 0.01$, compared with normoxia control groups, # $p < 0.05$, compared with hypoxia control groups.

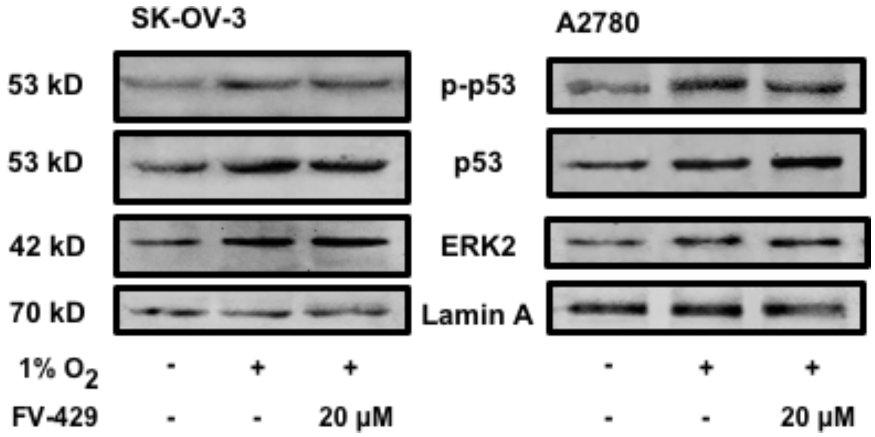

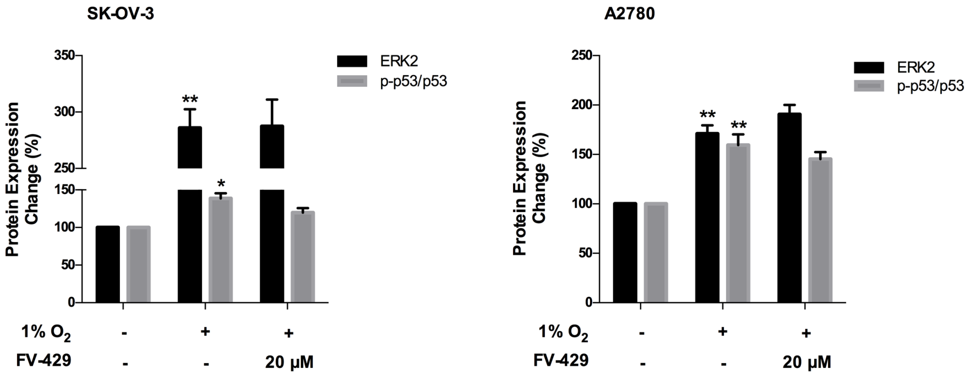

Fig. S6. The influence of FV-429 on ERK2, p-p53 and p53 expression in nucleus under hypoxia. After 24 h-treatment of FV-429, the expression of ERK2, p-p53 and p53 in nucleus was detected by western blots. Protein expression change was represented by densitometric analysis. The results are representative of three independent experiments and expressed as Means ± SD, * $p < 0.01$ and ** $p < 0.01$, compared with normoxia control groups.

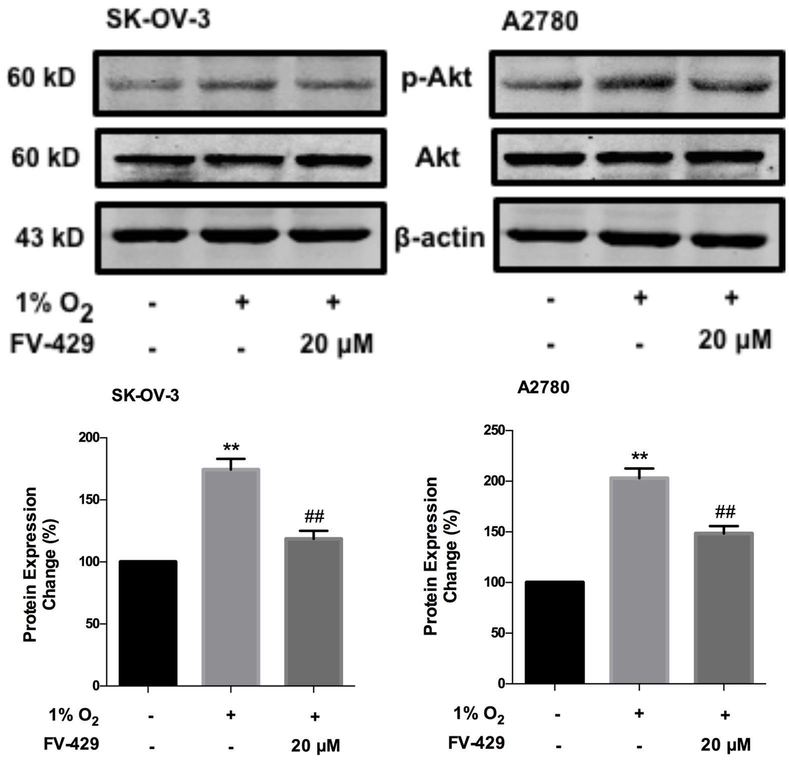

Fig. S7. The influence of FV-429 on p-Akt and Akt expression under hypoxia. After 24 h-treatment of FV-429, the expression of p-Akt and Akt was detected by western blots and p-Akt/Akt ratio was analyzed. Protein expression change was represented by densitometric analysis. The results are representative of three independent experiments and expressed as Means ± SD, ** $p < 0.01$, compared with normoxia control groups, $^{\#\#}$ $p < 0.01$, compared with hypoxia

control groups.
